# Malaria’s contribution to World War One – the unexpected adversary

**DOI:** 10.1186/1475-2875-13-497

**Published:** 2014-12-16

**Authors:** Bernard J Brabin

**Affiliations:** Clinical Division, Liverpool School of Tropical Medicine, Pembroke Place, L35QA Liverpool, UK; Global Child Health Group, Academic Medical Centre, University of Amsterdam, Amsterdam, Netherlands

**Keywords:** World War One, Malaria, Quinine

## Abstract

Malaria in the First World War was an unexpected adversary. In 1914, the scientific community had access to new knowledge on transmission of malaria parasites and their control, but the military were unprepared, and underestimated the nature, magnitude and dispersion of this enemy. In summarizing available information for allied and axis military forces, this review contextualizes the challenge posed by malaria, because although data exist across historical, medical and military documents, descriptions are fragmented, often addressing context specific issues. Military malaria surveillance statistics have, therefore, been summarized for all theatres of the War, where available. These indicated that at least 1.5 million solders were infected, with case fatality ranging from 0.2 -5.0%. As more countries became engaged in the War, the problem grew in size, leading to major epidemics in Macedonia, Palestine, Mesopotamia and Italy. Trans-continental passages of parasites and human reservoirs of infection created ideal circumstances for parasite evolution. Details of these epidemics are reviewed, including major epidemics in England and Italy, which developed following home troop evacuations, and disruption of malaria control activities in Italy. Elsewhere, in sub-Saharan Africa many casualties resulted from high malaria exposure combined with minimal control efforts for soldiers considered semi-immune. Prevention activities eventually started but were initially poorly organized and dependent on local enthusiasm and initiative. Nets had to be designed for field use and were fundamental for personal protection. Multiple prevention approaches adopted in different settings and their relative utility are described. Clinical treatment primarily depended on quinine, although efficacy was poor as relapsing *Plasmodium vivax* and recrudescent *Plasmodium falciparum* infections were not distinguished and managed appropriately. Reasons for this are discussed and the clinical trial data summarized, as are controversies that arose from attempts at quinine prophylaxis (quininization). In essence, the First World War was a vast experiment in political, demographic, and medical practice which exposed large gaps in knowledge of tropical medicine and unfortunately, of malaria. Research efforts eventually commenced late in the War to address important clinical questions which established a platform for more effective strategies, but in 1918 this relentless foe had outwitted and weakened both allied and axis powers.

## Background

**Figure 1:**
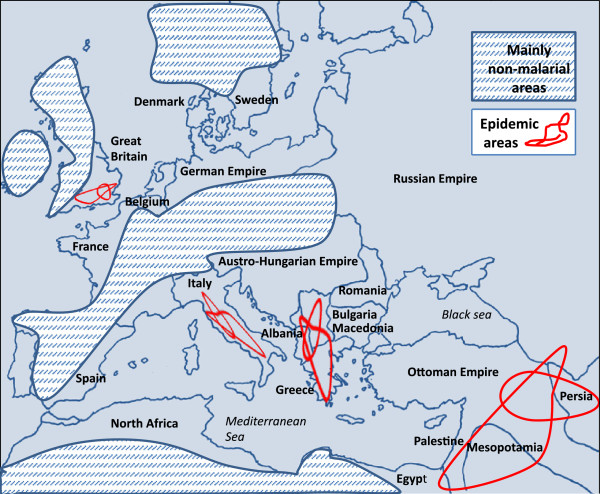
**Geographic distribution of malaria transmission in theatres of the First World War.** Source: references [3] and [4], modified to illustrate non-malarial areas. Epidemic areas are schematically drawn and indicative of regions affected.

An unexpected adversary in the First World War was malaria. It attacked all combatant armies, with adverse consequences for vast numbers of troops, and devastated large civilian populations as a result of the environmental, civil, and demographic effects of troop dispersions and activities. The term 'war malaria’ describes this paradigm [1], and captures the fact that malaria control in peace-time differs from that in war. This difference was exaggerated at the beginning of the First World War because military personnel were unaware that malaria was so widespread through many parts of Western and Eastern Europe (Figure 1). Although historians have recognized that a high proportion of troops died from disease rather than combat [2], malaria has received little emphasis, such that the subject is frequently not indexed in historical books or referenced in First World War manuscripts.

By 1914, parts of Europe had made considerable progress in malaria control, particularly in Italy and Greece [5, 6] which was a major achievement considering that, around 1900, the anopheline mosquito cycle was only just becoming accepted, and the important vectors, as well as the developmental cycle in the red cell, were only recently recognized. The damage the War inflicted on the political, social, and economic progress achieved in malaria control lasted until well into the twentieth century. There are questions as to why the First World War was so conducive to an explosion of malaria, leading to epidemics, conflicts on solutions, and clinical controversies on how to manage the basic disease in individual troops in different malaria endemic settings. These issues affected all armies as the malaria parasite had no alignment and could be transmitted unseen across theatres of war. The activities of soldiers aided its progress by increasing mosquito numbers through operational activities favouring transmission, especially as soldiers tended to burrow underground which is conducive to water – logging and favourable to mosquito breeding. By 1916, as more countries became involved, these effects multiplied creating ideal war conditions for the outbreak of several European malaria epidemics. The consequences compromised military planning, logistics, and the battle prospects of combatants, who became increasingly ineffective, as well as affecting huge swathes of civilian populations [6]. Re-deployment of non-immune troops to replace sick troops in malaria-affected war zones enhanced the problem, as did the inability to effectively treat soldiers suffering from malaria. Deployment of large numbers of troops from tropical Africa and India introduced into the European theatre a large human reservoir of infections with new parasite strains carried by semi-immune soldiers. The lack of knowledge of the hepatic stage of development of the *P. vivax* malaria parasite, which was only described in 1948 [7], compounded the situation and was instrumental in recurrently ineffective treatments being prescribed, and evacuations of vast numbers of sick troops from combat zones.

Only late in the war was it appreciated that preventive measures were possible. In the interim medical doctors struggled against this clinical adversary. The war documentation on the topic is diverse and has not previously been assimilated. Much reporting on the size of the problem has been anecdotal, due to limitations in surveillance statistics. This review aims to summarize available information and contextualize the main aspects in order to establish the nature of this formidable adversary in times of war.

### Search strategy

**Figure 2:**
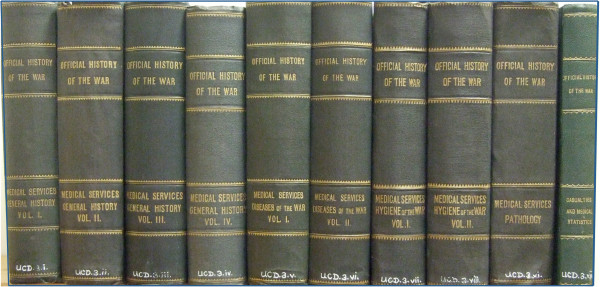
**Official UK Government War Volumes on Medical Services, Hygiene, Pathology, and Medical Statistics.** References: [2, 8–14]. Volumes on Surgery of the War also published [15].

Physical searches for historical information used library resources at the Liverpool School of Tropical Medicine, and the inter-library loan facilities of the University of Liverpool. Journal references in English, French, German and Italian were searched for the years 1914–1925. The Historical Library Collection of Tropical Medicine at the Liverpool School of Tropical Medicine and the Ronald Ross Archive at the Sydney Jones Library, University of Liverpool, were searched. For recent publications electronic databases were examined for historical context and scientific content. These included PubMed, Scopus, ISI Web of knowledge, the UK Parliamentary Records Resource, The Guardian newspaper and The Times newspaper Histories of the War. Reference lists from both general medical and historical journals were searched. Lack of appropriate indexing terms in early publications restricted the search for articles written before availability of electronic databases. The Official UK volumes published by His Majesty’s Stationary Office on Medical Services, Hygiene, Pathology, Casualties and Medical Statistics of the War were an important resource and are page referenced, as information on malaria was widely indexed across all volumes [2, 8–14] (Figure 2).

### Military malaria surveillance statistics 1914–1918

Initially it is necessary to estimate the military populations at risk, and associated malaria related mortality. Central to estimates of malaria exposure risk is information on troop numbers in the exposed military populations, which is data largely available in historical references and archives. Officially reported annual strengths of the British Expeditionary Forces in all theatres of the War were published in 1931 [2]. Figures are also available from historical sources that take account of troop movements and re-deployments. Few statistics are available for 'followers’, or attached military personnel, who were not fighting troops. Table 1 provides information of military populations exposed for both the allied and axis powers. This includes regional areas of troop locations. Also provided are malaria surveillance case numbers, and malaria incidence estimates per 1,000 troops for the periods specified in the table. When publications included numbers of deaths related to malaria, this information was used to estimate case fatality, which is as a range when sources giving variable mortality estimates were available. Estimates of the approximate military populations exposed are provided taking account of their variable composition. Troop numbers are often provided on an annual basis, which would include many of the same troops tallied annually in sequential years. In terms of malaria risk per annum these numbers should be interpreted additively, ie 100,000 men present over four years in a military zone equate to a total of 400,000 persons years malaria risk for the time period of the war. Another problem is that, although reference sources are listed in the table, there are differences in published troop numbers between sources. For this reason ranges of troop numbers are provided. This table is the first compilation of global malaria troop statistics for the First World War. It is divided according to the regional locations of troops of both allied and axis powers located in Europe and the Middle East, or outside this area in sub-Saharan Africa. In the European theatre there is the Western front in France and Belgium bordering with Germany; the large Eastern Front which stretched across the Austro-Hungarian border with Russia and Romania; a more Southerly front in the Balkan region of Macedonia including borders with Greece, Bulgaria, Albania and Turkey; an Eastern Front of the Ottoman Empire borders in the Middle East including Syria, Palestine, as well as Egypt, and Mesopotamia (modern Iraq). In sub-Saharan Africa locations are listed in East, West and South-West Africa. Allied armies were: Great Britain, France, Italy, Russia, Serbia, Romania and Albania. Central axis armies were: Germany, Austro-Hungary, the Ottoman Empire, and Bulgaria.

**Table 1:** **Military malaria surveillance statistics 1914 – 1918**

Military forces	Location	Years	Military case (n) and incidence estimates	Military deaths (n)	Case fatality, %	Military population exposed (n)	Malaria species ^a^	References
*Europe and Middle East*								
British	Macedonia and Salonika plain	1916 (Oct) -1918	162,517, 369 per 1000^c^	787	0.2-0.48	404,207^b^ – 549,111	Mostly Pv	[16], ([2] p2, p80),
						British, French 150,000, Serbs 200,000		[1, 17]
French	Macedonia	1916–1917	66,271	667	2.10^d^			[18–21]
British	North Russia	1918–1919	35, 8.7 per 1000, 32,688	?		22,258	Pv, Pf	([2] p2,p80)
German, Bulgarian	Balkans	1915–1918	113 per 1000		–	380,000 - 462,000	Pv	[22, 23]
	Macedonia	1916-1917	21,672	?	0.92	–	Mostly Pv Pf, Pm Pv, Pf	[19, 22]
British, French	Italy	1917-1918	7373 (40% relapses)	?	–	145,764 - 210,943	Pv, Pf	([2] p2,p80),
Italian		1915-1918	Many thousands	?	0.03-1.3			[6, 12]
Australian Desert Mounted Corps	Palestine	1918 (May-Dec)	Of 19,652 illnesses, 6,347 slide positive			40,000	Mostly Pf	[24]
	Palestine	1918 (Sept-Oct)	1940	98	5.05^e^			[24]
British & Dominion	Egypt and Palestine	1914–1918	40,144	854 ^f^	2.13	1,192,511	Pv, Pf	([2] p80), [24]
British	Palestine	1918 (Apr-Sept)	7,270			70,000		[25]
Turkish	Ottoman territory	1914-1918	412,000 - 461,799	20,000 -23,351	4.8	350,000	Pv, Pf	[26, 27]
German	Ottoman territory	1915-July 1918	4,763, 308 per 1000	?	–	5540		[22], ([28] p231)
German	Dardanelles	Apr 1915-Jan 1916	200 per 1000^g^	?	–	1000		([28] p231), [29]
Mixed allied force	Mesopotamia	1914-1918	59,323	284 ^h^	0.76	889,702 – 969,388	Mostly Pv	([2] p2 p80)
	Kuwait, Nasiriya	1915–1917	1,365, main admission	?	–	468,987 - 489,000		[10]
	Dardanelles	Apr 1915-Jan 1916	1,473	5	0.34			[1], ([2] p80)
Western Front: Allied & United States	North-East France and Belgium	1914-1918	9,022, outbreaks of <20	14	0.16	5,399,563 - 6,843,563	Mostly Pv	([30] p75,p115), ([2] p80), [31], ([32] p439-441)
Western Front: German		1914-1918	11,222, <1 per 1000	?	–	5,200,000	Pv, Pf	[22], ([32] p439), [33]
Eastern Front Allied: Russian, Romanian	Russia, Romania, Armenia, Georgia. Anatolia, Albania	1915-1916	Albanian armies suffered severely from malaria	?	–	525,000 ^i^	Pv, Pf, Pm	([30], p153),
								[21, 34, 35]
Eastern Front Axis: German	Galicia and Anatolia	1914–1918	29,952	?	? ^k^	228,000 – 750,000^j^		[22, 28] p137, [35]
			5.1 per 1000					
Turkish, Austro-Hungarian	Yugoslavia	1917-1918	128 per 1000	?	–	270,000 – 1,045,050		([30] p47), ([36] p399)
Evacuations to England from Europe and Africa	UK	1917-1918	34,000, 500 local cases in Southern England	323	0.95	≥34,000	Pv, Pf	([17] p140), [37]
*Outside Europe and Middle East*								
British expeditionary	East Africa, Kenya, Tanganyika	1915-1918	145,850 troops and 68,914 indigenous, 51.000 hospitalized, 126 per 1000(troops), 20 per 1000 (others)	831 troops with 2839 indigenous	0.57	250,000 British (75,366) and indigenous with 150,000 Indian	Mostly Pf	[2] p2, p80, [39]
					4.12			
	South-West Africa	1914–1915	518	2	0.39	33,000	Mostly Pf	([2] p2, p80)
	Cameroon	1915–1916	2410	5	0.21	3000	Pf	[14, 40]
	Sierra Leone	1914	401, 1839 per 1000	? 0	? 0.00	218	Pf	[41]
German expeditionary	Togo, East and South-West Africa	1914-1918	Lower incidence as mostly indigenous forces used	?	–	18,000 ^l^, 3000 German troops, remainder indigenous	Mostly Pf	[35, 42]
USA naval	Americas, Caribbean	1917-1918	4,746 hospital cases, 68,373 man-sick days	7	0.15	–	Mixed	[43]

Dashed indicate information not identified, and question mark unknown mortality case numbers.

^a^Pv: *Plasmodium vivax*; Pf: *Plasmodium falciparum*; Pm: *Plasmodium malariae*.

^b^Estimate includes British, French, Serbian, and Russian troops.

^c^13.7% in British troops; 27.4% in Indian troops.

^d^Of 100 autopsies of influenza pneumonia, 83 showed definite evidence of previous malaria [19].

^e^Estimated from graphic data from all cases in Sept-Oct and deaths reported in October [24].

^f^Deaths due to *P.falciparum* infections. Confirmed diagnoses in 32 of 67 autopsies; many complicated by dual infection with influenza bronchopneumonia [44].

^g^Estimate for German troops only.

^h^Deaths for years 1917–1918 only.

^i^Brusilov offensive 1915–1916.

^j^Russo-Turkish offensive winter 1915 – 1916.

^k^Of 2,873,000 men mobilized on the Turkish side in Ottoman territory, approximately 466,759 died of disease [28].

^l^Estimate for East African German forces.

War-time statistics may be underestimated by health officials as some armies did not keep adequate records, and for several locations there is missing data, especially for mortality, and for civilians who were exposed to many of the same adverse conditions. One previous approximation of the number of allied troops with malaria was 500,000, although the basis for the calculation was unclear [30, 37]. Incidence estimates per 1,000 men were available for some, but not all, locations. The sum total of case numbers for the War for the allied troops is 617,150 with 3,865 malaria deaths, which gives a case fatality estimate of 0.63%, or slightly more than one in every 200 infected soldiers dying from malaria. For the axis powers these estimates are 562,096, with 23,351 deaths, which gives a much higher malaria case fatality estimate of 4.15%, or more than four deaths for every hundred soldiers with malaria. This difference could reflect better management practices, and quinine regimens used by the allied forces in cases of severe malaria, or the influence of pre-War prevention measures developed by Ross on malaria control during the War. The table also lists the reported malaria case fatality estimates specific to different campaign areas. These vary between 0.15% - 5.05%. This variation partly arises due to troop composition, as regiments with larger contingents of tropical indigenous troops would be expected to have lower mortality risk due to their acquired immunity. Another factor is the proportional exposure risk in different areas for *P. falciparum* malaria compared to *P. vivax.* This data refers to the direct impact of malaria, but does not account for indirect effects on morbidity and mortality. For example soldiers anaemic with chronic malaria would succumb more readily following battle injuries or major surgery.

Table 1 indicates evidence for the intensity of malaria in several locations, particularly in Macedonia (present day Greece and environs), The Ottoman Empire (present day Turkey and environs), Mesopotamia (present day Iraq and Iran), Palestine, and East Africa. Malaria was a smaller problem on the Western Front, although the destruction of dykes in Flanders, causing local flooding, led to indigenous *P. vivax* cases. Prisoners of war returning to Flanders, who were infected in German concentration camps, is also reported [31]. In trenches where both European and African troops were present a small number of *P. falciparum* cases were reported amongst German troops [33]. In Gallipoli, malaria was never reported as a prominent cause of inefficiency, although the coastal area of the peninsula was considered highly malarious.

Malaria had a much greater impact on the Eastern Front involving the Russian, Romanian, and Albanian Troops, although quantitative information on malaria is not adequately defined for these countries. These armies were probably severely affected, with substantial malaria–related mortality [34]. Statistics shown in Table 1 for the German armies were reported by Zieman [22], although these did not provide mortality data, and he indicated they were underestimates. The accuracy of estimates of troop numbers resident in the Ottoman Front have been questioned [36], and Erickson considered that earlier estimates vastly underestimated the true number of Ottoman casualties from disease as well as combat [28]. By the end of the Brusilov offensive in late summer 1916, the Hapsburg army had officially lost 464,382 men and 10,756 officers, with 350,000 prisoners of war - a loss from which they never recovered. High malaria mortality estimates (23,000 approximately) have been reported for Turkish troops [26], but the number of cases and deaths for the army as a whole is difficult to quantify with no statistics for prisoners of war who would be likely to receive little in the way of protection from infection.

Underestimation in surveillance statistics is difficult to quantify due to failure to identify low grade or asymptomatic infections, which can cause debilitating chronic anaemia without occurrence of febrile episodes. The statistics also do not reflect the occurrence of relapsing vivax infections in soldiers taking low doses of prophylactic quinine, or falciparum infections in soldiers from the tropics with partial malaria immunity. Diagnostic misclassification would be expected unless a blood slide was taken, as clinical features of acute malaria have a broad differential diagnosis that includes diarrhoea and pneumonia. Febrile soldiers may also have suffered mixed malarial infections [45]. Autopsies conducted in Palestine by Manson-Bahr confirmed mixed infections with bronchopneumonia and malignant tertian malaria (*P. falciparum*), with influenza as dual infection. He considered that the mechanisms of early diagnosis and treatment broke down in rapidly advancing troops in malarious areas [44].

Use of regional malaria incidence data from Table 1 and the corresponding military population estimates allows the expected number of malaria cases in the military to be calculated for a given region (Table 2). Comparison of the expected and observed estimates shows some agreement except for the Ottoman territory and East Africa, where incidences are almost certainly underestimated, and do not take sufficient account of recrudescent or relapsing infections, which would greatly increase expectant numbers. This suggests that at least for these areas incidence rates underestimate the true rates. Malarial mortality due to Blackwater Fever was reported amongst expeditionary forces in East Africa [12], although there was no record of this occurring amongst black African troops. Of German troops in East Africa who died other than from wounds, 64.2% died from Blackwater Fever between 1914 and 1917. In Macedonia, of 136 cases of Blackwater fever, 36 died (26.5%), and amongst the French, mortality was 30% [12]. It was reported less frequently in Togoland and Mesopotamia. In Macedonia, Wenyon considered that hardships, exposure and unpreparedness under war conditions explained the high case fatality from malaria in 1916 (1.01%), and that the lower mortality in 1917 and 1918 (0.37% and 0.31% respectively) would have been still further reduced if ordinary peace-time precautions had been available [19]. High mortality was due to life under war-time conditions and not any particular feature of the parasite, although it followed closely the incidence rate of falciparum malaria. Overall British mortality from malaria in Macedonia was 0.7 per 1,000 troops, while for the French, for the same period (January-June 1918), mortality was ten times higher (6.4%) [19].

**Table 2:** **Estimated malaria cases based on size of military forces**

Location	Period ^a^ (years)	Malaria incidence (per 1000 troops)	Estimated military population	Expected number of malaria cases ^b^	Observed number of malaria surveillance cases
*British and Dominion forces*					
Macedonia	3	369	404,207	149,076	162,517
Western Front	4	<1	5,399,563	<5,400	9,022
			6,843,563^c^	<6843	
East Africa	3	126	250,000	31,500	145,850
North Russia	2	8.7	22,258	194	35
*German and Central Powers forces*					
Balkans	4	113	380,000	42,940	32,688
			462,000	55,206	–
Ottoman territory:					
- Turkish troops	4	308	350,000	107,800	412,000 –461,799
- German troops	2	308	5540	1689	4,763
Dardanelles – German troops	1	200	1000	200	–
Galicia and Anatolia Eastern Front	2	5.1	228,000^d^	1,163	29,952
			750,000^e^	3,825	–
			1,045,050	5,330	–
Yugoslavia	2	128	270,000	34,560	–
Western Front	4	<1	5,200,000	<5,200	11,222

Dashes: information not identified.

^a^Approximate duration of exposure.

^b^Estimate based on single incident case per soldier, and does not include recurrent episodes or relapses for the same individuals.

^c^Expected malaria cases based on two estimates for Western Front troop sizes.

^d^Military numbers only for Russo-Turkish offensive winter 1915–1916 [35].

^e^Expected malaria cases using estimates for Hapsburg army size of 750,000[46] or 1,045,000 [32].

### Malaria epidemics during the war

A number of basic conditions cause epidemics [47], sometimes separately, or together as in the First World War. These occurred in the regions of Macedonia, Palestine and Egypt, and in Western Europe following the return of troops to England, Italy and elsewhere. An increase in vectorial capacity due to high production of vector anopheline mosquitos was probably critical. This could have resulted from more breeding places (or deterioration in larval control), prolonged mosquito survival, or increased man-vector contact. In desert fringe regions rainfall and malaria transmission are often connected. The El Nino Southern Climatic Oscillations (ENSO) for the years of 1914–1915 and 1918–1919 may have produced favourable climatic conditions for epidemic transmission as the impact of ENSO in malaria epidemic prediction is well-established [38]. The ENSO for 1918–1919 was one of the strongest in the twentieth century [48]. The movement of infected persons, as troops migrated from India and sub-Saharan Africa, combined with the immigration of non-immune European soldiers further created ideal conditions for the explosive epidemics which occurred.

### The Macedonian epidemic

**Figure 3:**
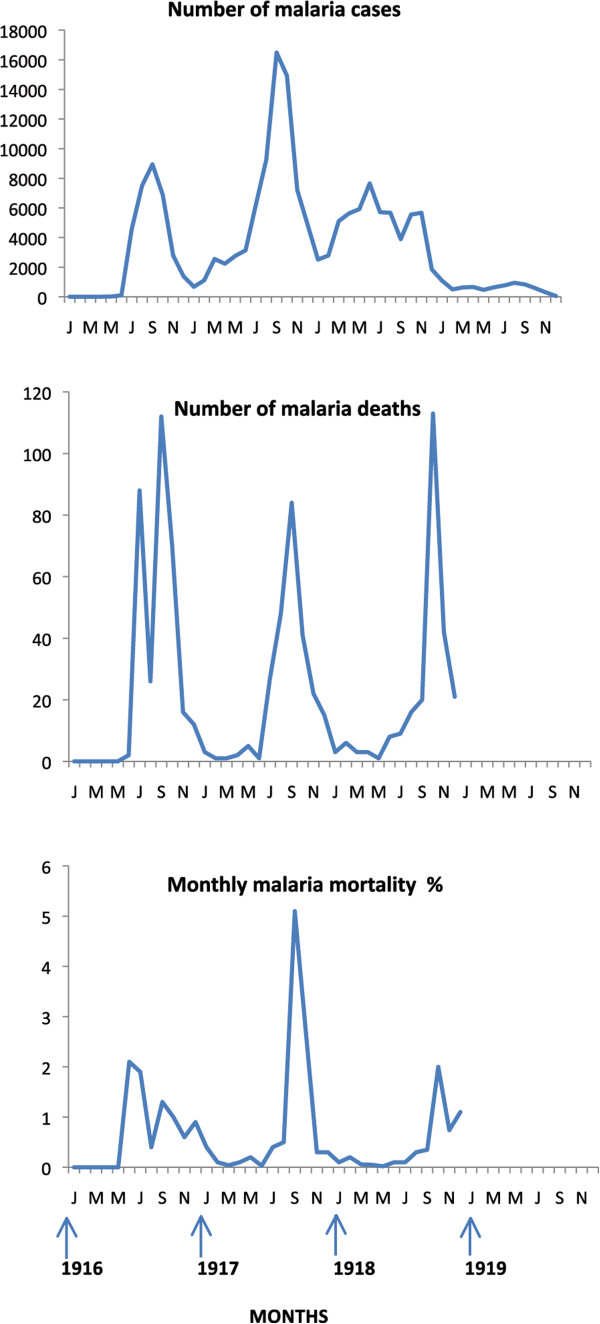
**Epidemic pattern of malaria in allied forces in Macedonia 1916–1919.** Source: references [10, 11]. Horizontal axis in months.

**Figure 4:**
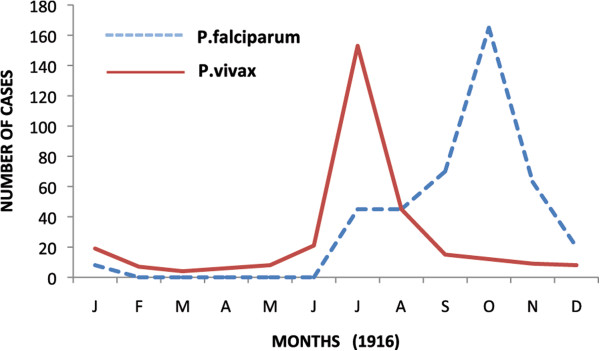
**Parasite species differentiation in French troops in Macedonia.** Source: reference [20]. Horizontal axis in months.

This event was one of the greatest medical surprises of the war with more than a quarter of the half a million allied troops immobilized with sickness and equivalent numbers of Bulgarian and German troops (Table 1). Amongst British troops, peak prevalence patterns were observed seasonally from July to November in 1916 and 1917, and with double peaks between May and November in 1918 (Figure 3). Relapsing vivax cases infected in the previous year may have contributed to the early peak prevalence in 1918, and falciparum malaria may have induced some vivax relapses in the late second peak in 1918 [49]. The species pattern observed amongst French troops in 1916 showed peak vivax prevalence preceding peak falciparum prevalence by three months (Figure 4) [20]. This was similar to the species pattern seen in British troops in Palestine and Egypt in 1918 [49]. No deaths were reported between January and March 1916, and epidemic peaks in mortality followed corresponding peak prevalence patterns (Figure 3), with mortality peaks in 1916 corresponding to the monthly peak prevalence periods of vivax and falciparum malaria. Mean malaria mortality remained below 2%, with peak mortality of 5.1% in September 1917.

**Figure 5:**
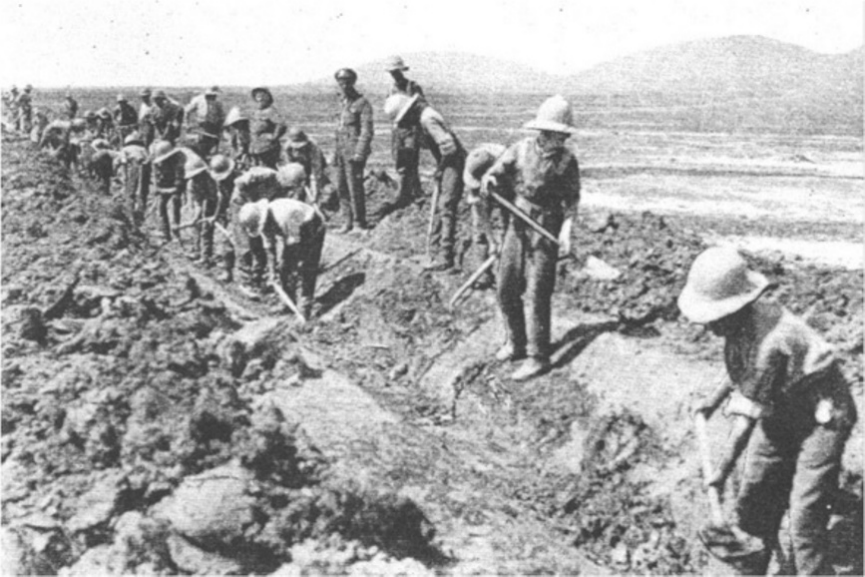
**Draining the marshes to prevent malaria on the Eastern Front.** Source: reference [52], published under the Open Government Licence, v 2.0.

The malaria epidemic in Greece resulted in ten allied casualties for every one inflicted by the axis powers [35]. There was no escape from the mosquito under the endemic conditions in which these armies fought. The surprise of the high mortality and the magnitude of the immobilized forces induced Generals to respond to the malaria crisis, which required a greater expenditure on labour to clear mosquito breeding sites. This effort was insufficient as it was not known that mosquitoes could spread beyond about 500 metres from their breeding sites. The main vectors were *Anopheles maculipennis* and *Anopheles superpictus,* and the sporozoite rate varied between 0.5% and 5.5% [19, 50]. The British, French and German armies were virtually immobilized for three years, with a French General telegramming that his army was in hospital with malaria [16, 18]. This caused French military authorities to think seriously about the crisis, hugely improved efforts at quinine prophylaxis of troops [18], and rapid evacuation of infected troops. As a result they experienced less malaria than the British. The British introduced the Y management scheme of quinine treatment (15 gr/day x 14 days, then 60 gr/wk x 6 days in optional divided doses; every soldier to keep 60 gr for self-treatment if relapses), which included removal of heavily infected individuals, with 25,000 men evacuated in 1917 [19]. Other measures included every known method of combating the disease including swamp and stream clearance (Figure 5), oiling, treatment of breeding places, fumigation, spraying, mosquito proofing and quinine prophylaxis. Despite these efforts, it is doubtful whether any appreciable reduction in infectious episodes occurred [19]. Most of these measures were introduced effectively too late in the campaign, partly due to conflicting opinions on prioritization, but also delays in obtaining netting and sufficient quantities of quinine [10]. Quinine prophylaxis had been recommended by Ronald Ross, consultant Physician to the Mediterranean Expeditionary Force, but was not favourably reported on by medical officers. Ross visited the area between 26th November 1917 and 18th January 1918, yet surprisingly makes no mention of the epidemic in his memoirs [51]. The area became a large military hospital and the spectacular image of a well-equipped army paralysed by malaria is a sober reminder of its potential severity.

### The Palestine and Mesopotamia epidemic

**Figure 6:**
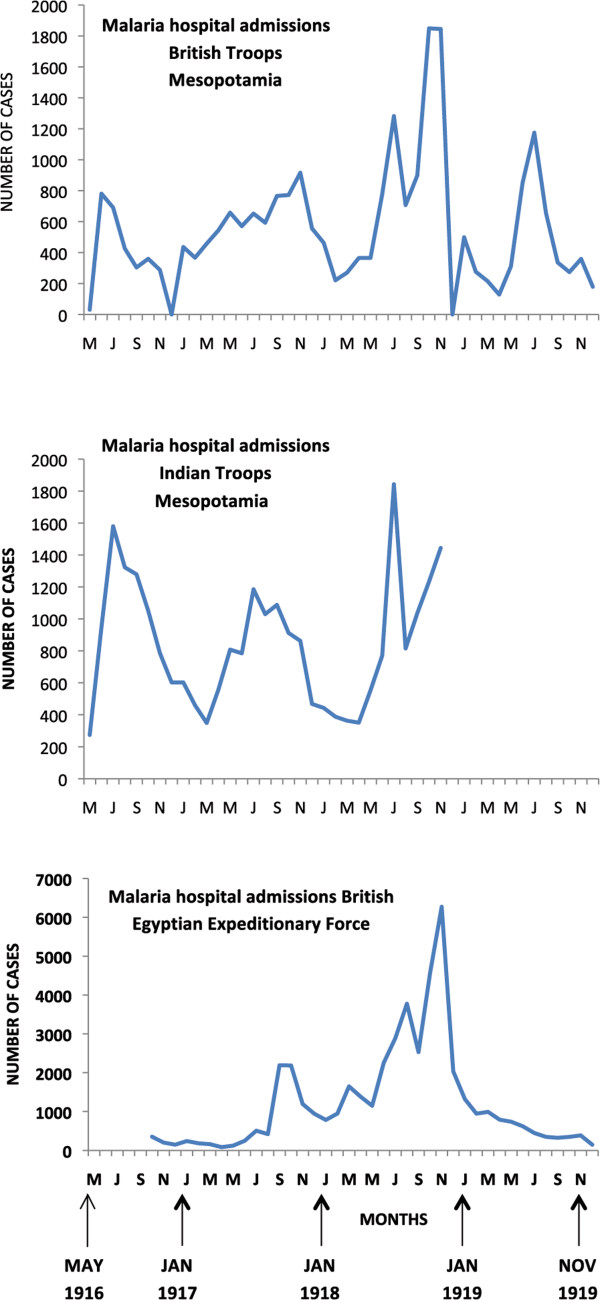
**Epidemic pattern of malaria in allied forces in Palestine 1917 – 1918.** Source: references [10, 11, 54]. Horizontal axis in months.

**Figure 7:**
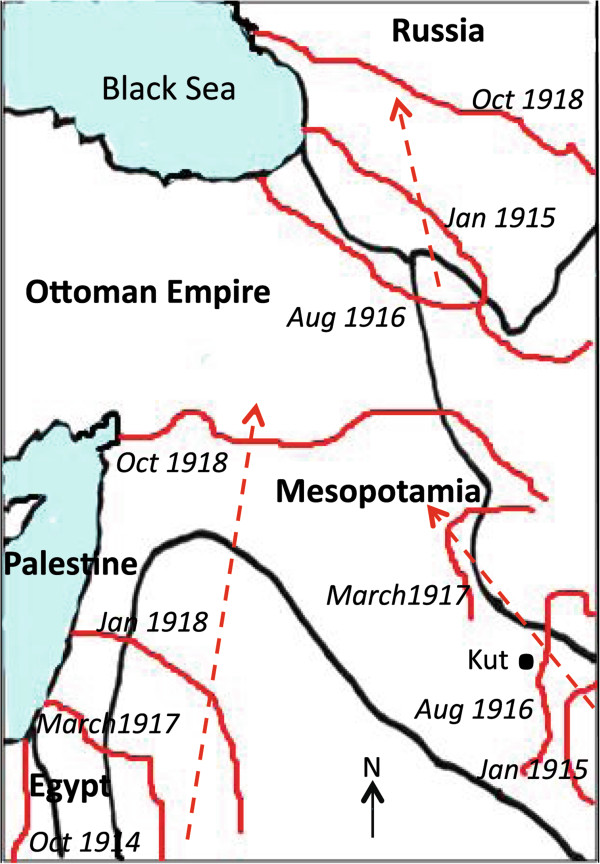
**Dynamics of troop movements in the Middle East, 1914–1918.** Source: adapted from reference [32].

**Figure 8:**
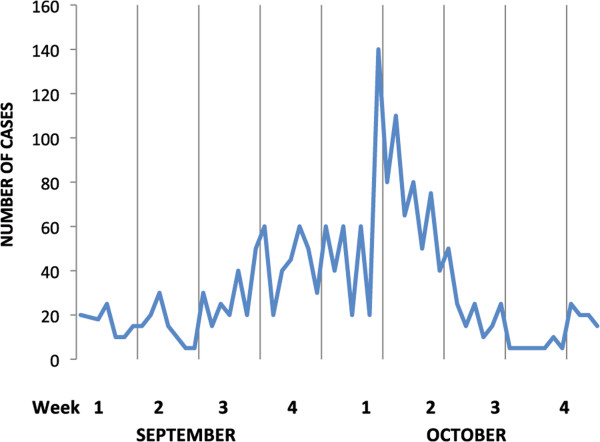
**Malaria epidemic ANZAC Mounted Division, Sept – Oct 1918.** Source: reference [24]. Case numbers estimated from graphical data.

The epidemic amongst British troops commenced immediately after they moved into this malaria endemic area in early 1916, and after the incubation period had passed (Figure 6). Malaria was also introduced into the Sinai region by troops from India, who eventually comprised about three quarters of troop numbers. It was difficult to locate camps away from highly endemic areas in southern Mesopotamia (modern Iraq.) Cases of marine malaria were reported with higher incidence in ships anchored closer to the shore [53]. Initially cases were mostly caused by *P. vivax* infection. Malaria became more widespread during the campaign with the largest epidemic occurring in 1918, with proportionally more *P. falciparum* infections. The operations of Indian and British forces in 1918 involved campaigns in some of the most malarious areas. Successive advancing fronts across the whole region continuously placed troops in new transmission areas (Figure 7). The British military failure at the siege of Kut in 1916 (Figure 7), and the overall military failure of the Turkish army was related to soldiers being overwhelmed by malaria, although the disabling of soldiers by the disease was not to the same extent as in Macedonia. Accurate returns on malaria were mainly available to the end of September 1918, and the main British military attack then occurred on September 19th [25]. The 1918 epidemic did not commence until temperature averages had risen above 15.5°C in early June, which were climatic conditions favouring mosquito breeding. If the British advance in mid-September had been delayed the successful outcome might have been different. Incidence was falling by November, which was considered due to the extent of the anti-malarial measures undertaken earlier in the year [25]. In addition to British and Indian troops, Australian mounted forces also experienced epidemic malaria with a high case fatality of 5%, which related to dual infections with *P. falciparum* malaria and influenza (Figure 8) [24]. Australian statistics on their cavalry advance showed that the sick outnumbered the battle wounded by 37:1 [49].

A small number of malaria diagnostic stations providing malaria microscopy were established in April 1918, by British and Australian commands, and with specially trained medical officers and orderlies [9]. Rapid diagnosis may have reduced case fatality and helped to check the epidemic in Palestine. Over 111,000 blood smears were examined by the Australian New Zealand Army Corps laboratory, and of 40,000 slides examined by British diagnostic stations 28.9% were positive [49, 55]. A number of other measures were introduced and by 1918 the extent of anopheline breeding meant there was no time to be lost. Large sums of money were spent on oiling and drainage, with £10,000 for drainage clearance alone [9]. Deep shell holes rapidly fill with water, and British clearance work was hampered by Turkish shelling of working parties. Some tributary origins were within Turkish lines and the British felt that the Turks failed to appreciate that the destruction of mosquito breeding places by the allies would also be beneficial to their own army as mosquitoes traversed front lines [25]. As a consequence, malaria amongst allied troops increased in frequency with further advances into Turkish positions (Figure 7). The micro-epidemiology of malaria in this varied terrain was challenging, and the operations were difficult and costly to sustain. The full effects of these anti-malarial measures are difficult to assess, and they were introduced late in the campaign. The drafting of replacement troops to Palestine from Macedonia introduced an additional human reservoir for mosquito infection, which reduced benefits achieved from the evacuation of infected troops.

### The epidemics in England and Italy

**Figure 9:**
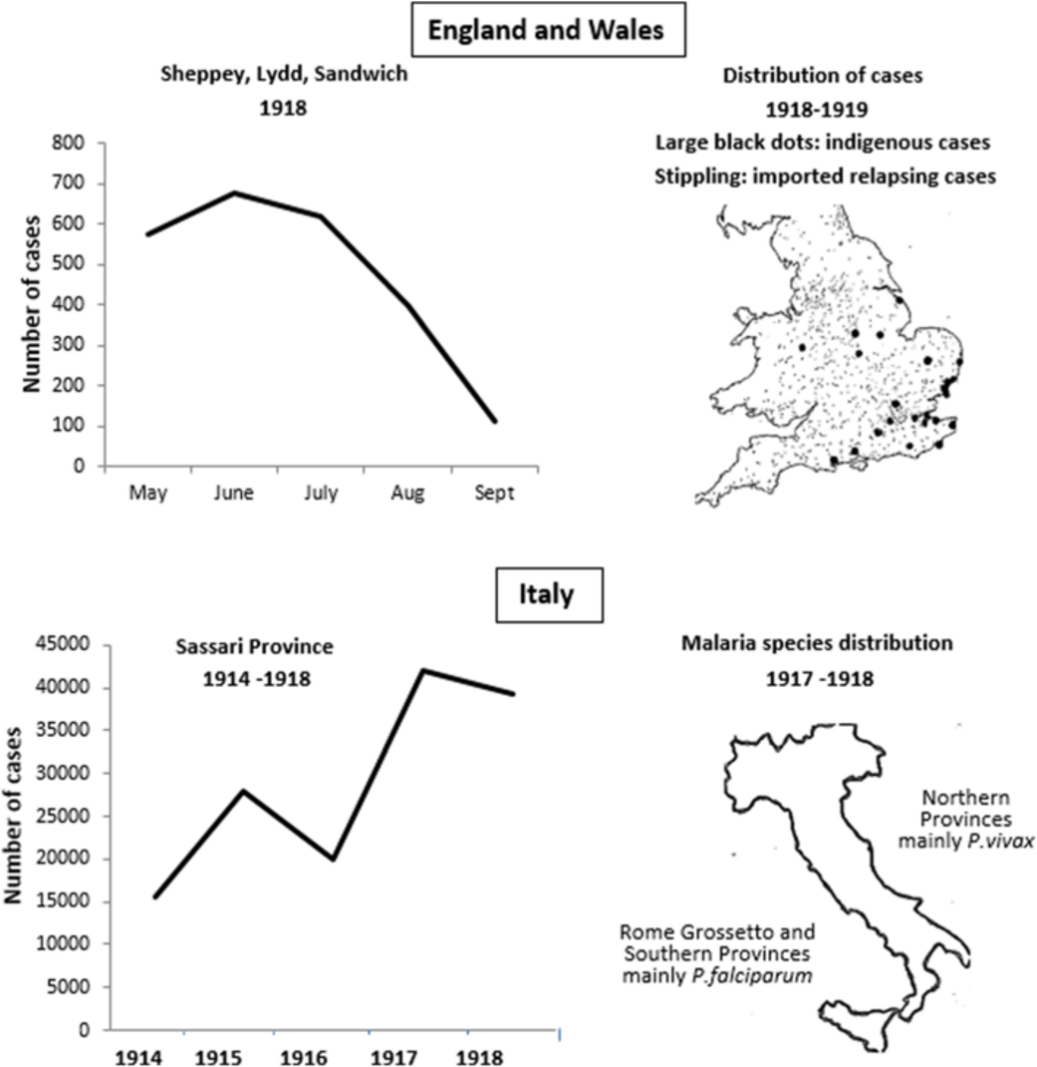
**Malaria patterns following troop evacuations in England and Wales 1917 – 1918, and Italy 1914–1918.** Source: references [6] and [60].

In England between 1900 and 1914 only two cases of malaria had been observed in soldiers who had never been out of the country [56, 57]. Following 1914, cases gradually increased reaching epidemic proportions in 1918 (Figure 9). In 1917, Ross had concluded that true indigenous malaria foci had never disappeared from the island of Sheppey on the north Kent coast, where healthy carriers of *P. vivax* were identified [58]. The Ministry of Health did not station soldiers returning from malarious regions in parts of Kent or Sussex because of concerns about local transmission. The First World War brought the problem of malaria back to England, leading to the last epidemic of malaria in the country and substantially increased local transmission (Figure 9). The problem arose from troops returning from Egypt, Turkey, Macedonia, Italy, West and East Africa. Table 3 shows malaria statistics on reinforcements to the British expeditionary Force arriving in France from Macedonia and Palestine in June 1918 and illustrates the magnitude of the problem [59]. These soldiers brought back infections of *P. vivax* and *P. falciparum* malaria to areas which had been free of disease for many years, and many had to be evacuated from France to England for treatment. Soldiers returning from a wide diaspora were admitted with malaria to the London Hospital during 1918 and 1919 (Table 4). Before the end of the war, 34,000 military cases returned to five camps set up in England for their treatment, and 500 locally transmitted infections were identified [60]. In 1919, the Ministry of Health made malaria notification compulsory [30] in order to identify infection foci.

**Table 3:** **Malaria statistics on reinforcements to British expeditionary Force arriving in France in June 1918** [59]

Regiment ^a^	% infected with malaria	Cases ^b^ (n)	Daily sick parade malaria statistics
5th Connaught Rangers	80	82	If regiment ordered to service in forward area at least 50% unable to comply
5th Royal Irish	75	80	30/650 parasite positive^c^
2nd Royal Dublin Fusiliers	100 (last 2 years)	175	25% parasite positive
13th Black Watch Scottish	80	573	30/day reported sick with malaria
14th Kings Liverpool	75	146	60-70/day with malaria
5th Inniskillin Fusiliers	75	175	600/824 have had malaria; 30% parasite positive
1st Kings own Yorkshire Light Infantry	70	42	Only 30% of regiment never had malaria
13th Manchester	80	52	Average 4/day relapsing
9th Gloucester	80	–	60-70/day with relapsing malaria
2nd Royal Munster	80	80	Men with acute malaria may not report sick
6th Inniskillin Fusiliers	75	21	110 hospitalized since June 1918
6th Leinster	75	87	480 with malaria since June 1st 1918
5th Royal Irish Fusiliers	75	58	27 reported malaria within 3 days of parade
7th Wiltshire	50	165	Less infected than other Battalions
4th Kings royal rifle corps	70	10	Only 30% not had malaria
2nd Northumberland Fusiliers	80	209	146 hospitalized en route to France with malaria. Average daily sick parade = 60
10th Camerons (Lovat’s scouts)	80	–	57/210 suffering relapses on day of inspection
3rd Royal Fusiliers	70	20	32 hospitalized en route to France
10th Black Watch	80	140	17/42 had malaria in morning sick parade
6th Royal Dublin Fusiliers	80	110	40 with malaria en route to France
12th Lancashire Fusiliers	80	56	33 arrived with malaria
Total	Mean 76.7	2281^d^	–

^a^Average Regimental strength = 800 men per regiment.

^b^Cases with diagnosis confirmed by microscopy.

^c^Blood smear positive on microscopy.

^d^Mean 15.0% parasite positive ( 2281/15,200, with average regimental strength of 800 men).

**Table 4:** **Malaria cases admitted London General Hospital February 1917 – January 1919** [61]

Location of origin	Admissions	***P.vivax***	***P.falciparum***	Mixed Pv and Pf
	(n)	(n)	(n)	(n)
Salonika	1686	746	82	20
East Africa	1049	496	43	26
India	126	35	2	–
Egypt	86	29	3	–
Palestine	63	11	3	–
Mesopotamia	46	19	–	–
West Africa	35	4	2	–
France	48	15	–	–
South Africa	25	4	1	–
Italy	25	7	5	–
Singapore	16	4	1	–
North America	11	3	–	–
Total	3216	1373	142	46

In a military camp on the Isle of Sheppey soldiers in training who had never left England contracted malaria, and in one regiment 30 cases occurred. These were locally contracted as *An. maculipennis* mosquitoes could be found without difficulty in occupied huts [60]. In the ports of Liverpool, London and Bristol, during 1917, more than 500 cases of malignant tertian malaria were introduced mostly from West Africa. The newly completed building of the Liverpool School of Tropical Medicine was nominated for use for hospital treatment of infected soldiers and undertook clinical trials of quinine treatment [62, 63]. The main source of infection for most naval and military cases, and nearly all civilian cases, which occurred after 1916, was relapsing vivax malaria among returned soldiers [64, 65]. Prompt identification of infection foci, and quarantining infected soldiers and sailors in a small number of dedicated hospitals, enabled effective epidemic control and limitation of indigenous transmission [65].

There was a rise in reported cases in several other European countries due to malaria introduced by returning troops [30]. In Vienna, 3717 cases were reported in 1919, and epidemics occurred in Albania and Corsica, although in Germany it was not as serious a problem after the War. There were post-war epidemics in Ukraine and Russia, and an outbreak occurred as far North as Arkhangelsk ('Archangel’) in the arctic [2, 30]. In Russia 360,670 cases were reported in 1919, rising to 5,865,828 annual cases in 1924, when incidence reached 446 per 10,000 population with at least 600,000 case fatalities [30]. In Italy, there was a ferocious epidemic, which devastated the nation in 1917–1918, in which tens of thousands of civilians and soldiers perished (Figure 9) [6]. The Italian Front was as violent as the Western Front, and Snowden reports that the horrors of battle were so great that some soldiers purposefully avoided taking quinine in order to become malaria-infected and be evacuated – a response described as '*malaria defeatism*’ [6]. The large number of evacuated troops and prisoners of war, collapse of the pre-war Italian malaria campaign, requisitioning of Italian hospitals by Austrian forces, shortages of quinine, and war-time disruption left the Italian population at high risk of malaria infection. In 1917–1918, the epidemic was at its peak and lead to Italy experiencing the worst malaria epidemic of the twentieth century [6]. Almost 50,000 soldiers were repatriated from highly endemic areas and created an epidemic route through to Southern Italy, which experienced an excess of falciparum infections compared to Northern provinces.

### Malaria in sub-Saharan Africa

African expeditionary forces were at high risk for *P. falciparum* infection. British and German armies were constituted with a majority of indigenous or Indian troops who should have been less susceptible due to their acquired immunity [2]. Table 1 gives estimates of the proportions of these contingents, and shows that in East Africa, whilst malaria incidence was lower in indigenous and Indian troops, their total malaria mortality was higher as they were a much larger fighting force. Imperial troops and supporting bearers serving in East Africa had a death rate that exceeded any other theatre of war [66], and measured up to those in the great European bloodbaths [67]. The medical provisions for the African and Indian troops were poor, and they were exempted from the general order for quinine prophylaxis. The Germans, who were a relatively small force, had a higher ratio of porters to soldiers (7:1) than the allies (4:1), and also high standards of disease prevention. They nevertheless reported some deaths from Blackwater Fever from staff receiving one grain quinine daily as self-treatment [68]. Ocean Road Hospital in Dar es Salaam was requisitioned from the Germans by the British at the outbreak of the War and was used for treating British soldiers. Although some attempts at malaria control were introduced in 1914, including sanitary inspections and importation of anti-larval fish [40], General Smuts, who commanded the British forces, may have been negligent with regard to malaria control offering too little, too late [66, 69]. The numerous preventive approaches so carefully developed in Macedonia and Salonika were not attempted, as most reliance was placed on quinine prophylaxis. Quinine supplied to the Army and Navy was below indicated strength due to war profiteering, whereas specimens captured from the Germans (and of German manufacture) produced quinine toxicity or cinchonism with much smaller doses. In July 1918, a small outbreak in marines visiting East Africa was attributed to sub-standard prophylaxis due to this problem [70]. Germany subsequently used the research station at Amani in Tanganyika (now Tanzania) to produce its own quinine supply. Sub-standard quinine was an important reason for the resurgence of malaria in other regions during and after the War [5, 71].

In Cameroon and Sierra Leone in West Africa, smaller numbers troops were deployed compared to East Africa, and few military deaths from malaria were reported [40, 41, 72]. Malaria incidence was high, despite troops using mosquito nets and quinine prophylaxis, and many troops had repeated admissions with marked anaemia and debility [41]. Expert microscopy and treatment was available. Amongst reinforcements arriving from India to Muscat, on the southern Arabian coast opposite East Africa, 25% had splenomegaly [73]. The extent of infection in these soldiers was not appreciated and many returned to India on account of fever after residence for only a short period in battle zones.

### The Pacific region

In Pacific malaria endemic regions, the New Zealanders took German Samoa in August 1914, and the Australians German New Guinea in September [32]. The Japanese occupied the Marshall, Caroline, and Marianas islands the following month. Fighting in these regions, which possibly had the highest malaria vectorial capacity of any war zone, would have resulted in serious malaria-related illness, as their malaria control strategies were similar, or less developed to those used elsewhere. It was known that anophelene mosquitoes carried malaria but the identity of the local vectors had not been established [74].

### Malaria control approaches

The greatest experience of preventive measures was derived from activities in Macedonia, and these were applicable to all theatres of the War and could be classified as: drug prophylaxis and treatment, mosquito deterrents, personal protection, or mosquito destruction [11]. New approaches were developed during the War which advanced practical and operational strategies. These are summarized and compared with pre-1914 approaches in Table 5. The lack of pre-war scientific evidence on all of these control strategies was a major restriction. There was minimal data available on acquired immunity to malaria, as well as controversy in Germany before the War between Erhlich and Bordet on the nature of the immune response to infections [75]. During the War, operational evaluations of some prevention measures were developed, and the most feasible was testing quinine treatment and prophylaxis, as it was more readily controlled, follow-up was realistic, and outcome determinants were easily measured. In view of the extent of the variety of these war-time trials, data on quininization is covered in a separate section. Personal prophylaxis with quinine was chaotic with weak understanding of medicines in management, problems in drug delivery due to night operations, enemy bombs and attacks, and servicemen may also have hidden the drug to exchange for cigarettes [76]. The British questioned the use of quinine for prevention and placed more emphasis on preventing contact with mosquitoes. A survey of 129 officers on the utility of quinine by the War Office reported only four felt it was of definite value, ten of some value, and the remainder of no value [11].

**Table 5:** **Approaches to malaria control prior to and during World War One**

Pre – 1914	1914 - 1918	
*Prevention European theatre & British Dominions* [77–79]	*British* [9–11, 13, 17, 25, 30, 44, 66, 73, 80–83]	*French* [18, 30, 84]
- Systematic inspection and notification of cases	- Scientific evidence on control strategy required	- Preliminary study of epidemiological conditions [84]
- Protection against mosquito bites (portable nets)	- Specially trained medical officers and orderlies required [44]	- Appropriate housing
- Portable mosquito proof rooms	- Availability of malaria diagnostic units with microscopes [9, 44]	- Eliminating parasite reservoir
- Protection of hands and feet (boots)	- Sanitary sections in field Divisions to supply material for anti-mosquito work [82]	- Small anti-larval measures and measures against adult larva
- Medicinal skin protection (effect transient)	- Mosquito brigades (in Palestine)	- Quinine treatment and prevention
- Mosquito reduction (fumigation, traps, fish as larvicides, oiling, drainage, screening breeding water, filling pools, piping to prevent leakage)	- Frequent inspections of anti-mosquito work [82]	- Collective mechanical defences
- Detection, isolation and specific treatment of all infected soldiers [79]	- Entomological studies of anopheles species [17]	- Bed nets and mosquito protection [84]
- Prevention by treatment	- Recognition problem of mistaken diagnoses [44]	- Reduction of negligence and scepticism of preventive measures by authorities [18]
- Personal domestic hygiene	- Siting of camps and evacuation of areas [11]	- Reduction of contradictory instructions on quinine use to avoid chaotic use of quinine
- Quinine prophylaxis	- Instructions on use of mosquito nets and net design. ^45^Bivouac netted tents [17], (Figure 11)	- Urine inspection for quinine detection (Tarant’s reagent) to control prophylactic administration of quinine [18]
- Consider public prophylaxis with quinine (if troops contracting infection outside barracks)	- Mosquito-proof canvas huts [17]	- Malaria specialists as advisers [84]
		- *Australian* [24, 55]
- Repeated measurements (malaria surveys)	- Mosquito swats [17]	- Malaria diagnostic units with field laboratories [55]
- Keeping troops in non-malarial hill areas [79]	- Occasional use of pyrethrum sprays (Lefroy’s fluid) for mosquito control [30]	- Aggressive control of mosquito breeding along front lines [55]
- Imposition of fines for non-cooperation Legislation on engineering work	- Quinine prophylaxis recommended by Ross and Medical Advisory Committee, but unfavourable response from medical officers [10, 80]	- *German* [85]
		- High standards of disease prevention
*Prevention in German possessions* [86, 87]	- Drainage operations, although impossible in mountainous terrain, or where campaign was highly mobile (eg, Palestine) [25]	- Emphasis on quininization [85]
- Local conditions decided most important activity	- Indian troops exempted from use of mosquito nets on grounds they were unpopular, and they had acquired malarial immunity [66]	
- Quininization (simplest and cheapest)	- Pamphlets explaining dangers [17]	
- Supervised quinine therapy and quinine prophylaxis trusted to exterminate malaria even in very badly infected areas,	- Recruitment blood examination for selection of smear negative candidates (West Indies) [13]	
- Environmental mechanical protection	- Instruction of medical officers on spleen examination technique of Indian troops [73]	
- Personal anti-mosquito measures		
- Reliance on acquired immunity [87]		

**Figure 10:**
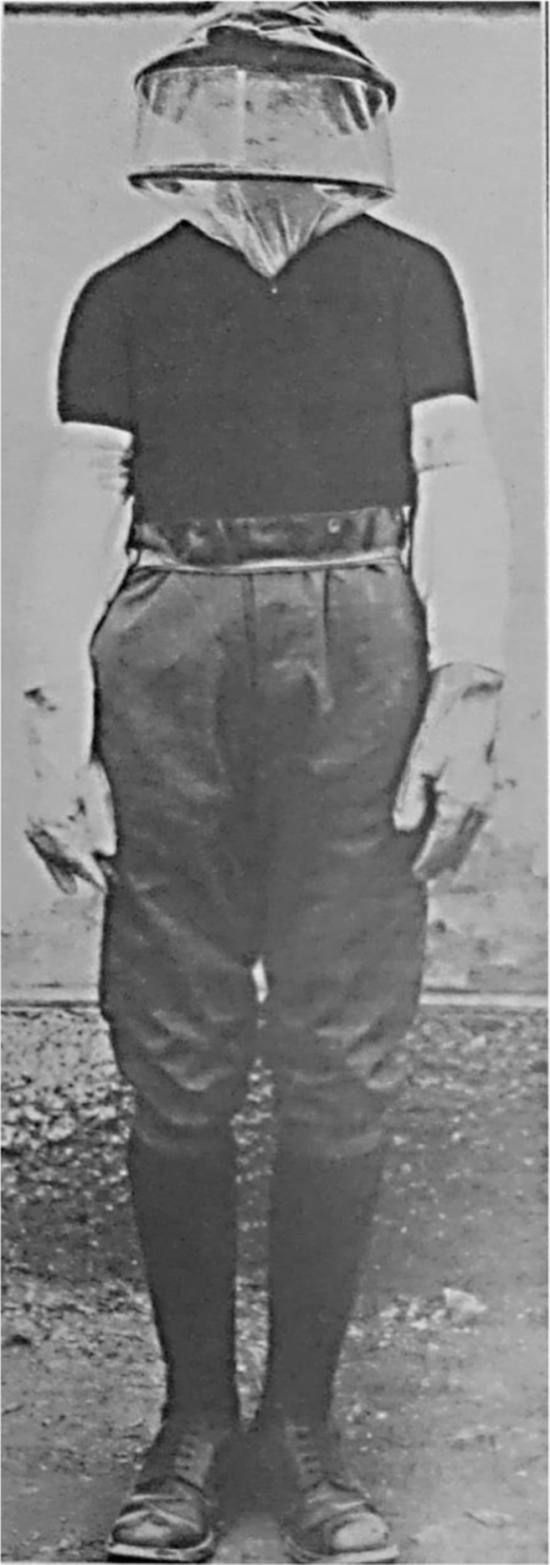
**Protective clothing for mosquito protection.** A general view of the special protective clothing in use. A head net is worn over a shrapnel helmet, and the special pattern shorts are turned down under the upper turn of the puttees. The gauntlet gloves are seen on the hands and forearms. (published under the Open Government Licence, v 2.0), [11].

**Figure 11:**
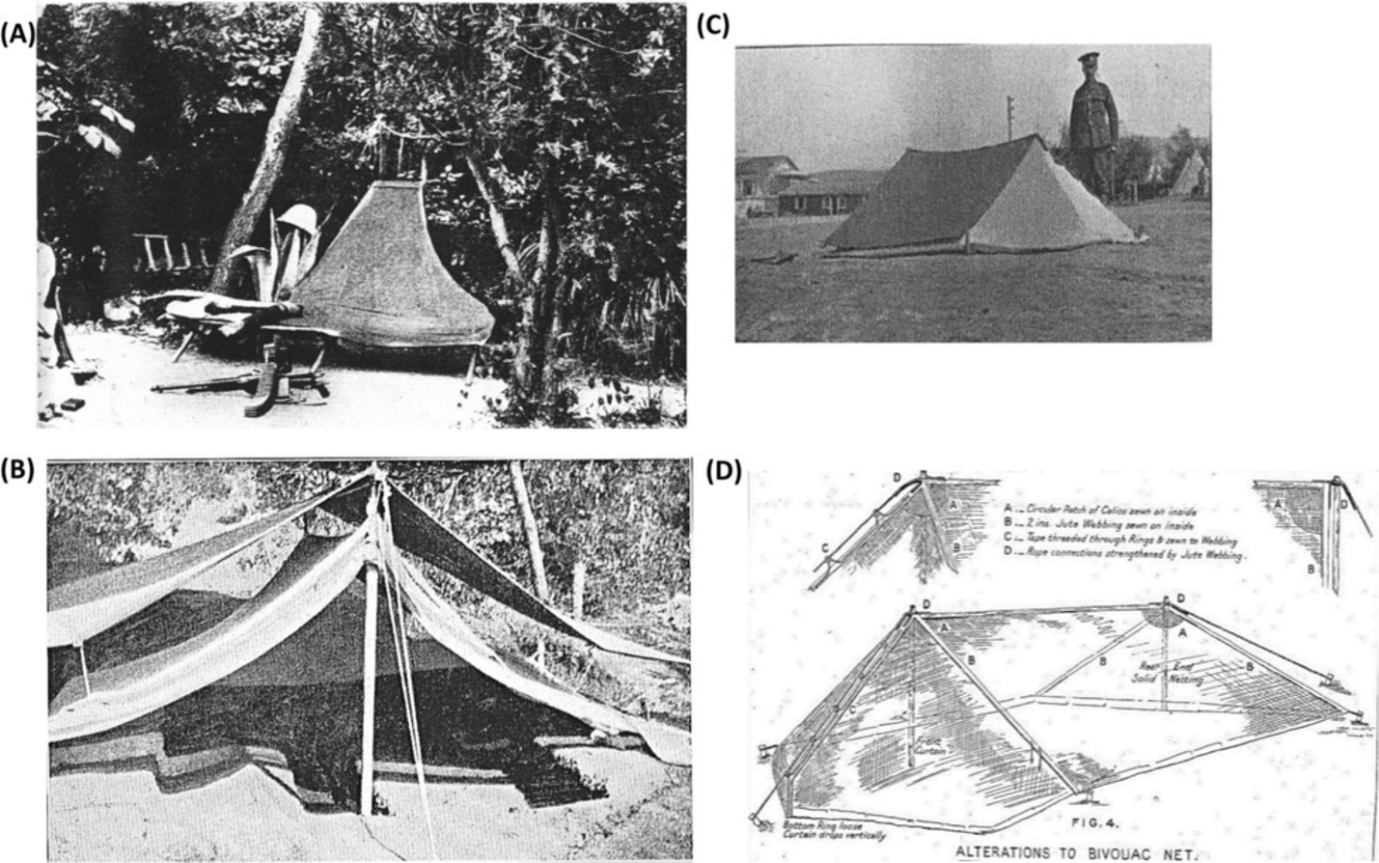
**Alternative military net designs for mosquito protection. (A)** Mosquito net with bell tent design as supplied to French soldiers in Salonika, Macedonia (source: The Times History of the War), [88]. **(B)** Bivouac net opened to show interior. The ledges forming beds are dug down to a depth of 9 inches and the trench for feet is dug down to a depth of 18 inches (published with permission of the Royal army Medical Corps Journal), [11]. **(C)** Modified Bivouac net supplied to British soldiers in Macedonia in 1918. The net is intended for two men and is covered by the two bivouac sheets. The front slides up and down the guy rope (published under the Open Government Licence, v 2.0), [19]. **(D)** Same net as in (c) showing alterations to improve protection (published under the Open Government Licence, v 2.0) [11].

Personal anti-mosquito ointments were messy and head nets and gloves were very restrictive especially in active combat (Figure 10). It was actually as important to protect infected men from uninfected mosquitoes as it was the reverse. Environmentally, the need for frequent spraying of pyrethrum for mosquito control deterred its use by German forces although the British army used it occasionally (Lefroy’s fluid) in the First World War [30]. In many areas, the very high density of mosquitoes made it impossible to control the vector. Extensive drainage was impossible in mountain terrain, or when campaigns were highly mobile. Identification of vectors gave a sounder basis for control and studies were undertaken on entomological parameters [50]. Notions of integrated control and the mapping of community reservoirs of infection were emerging and were completed in response to the epidemic in England [60], but remained notional in other areas. A large compendium of measures was required to tailor to the environment, although this was only really appreciated towards the end of the War. Negligence and scepticism with application of preventive measures were common and contradictory instructions were given by officers [18, 77].Late in the war, at least in Macedonia and Palestine, a considerable effort was placed on net design with the promotion of modified bivouac tents for rapid deployment (Figure 11). A small net for use by individual men was considered preferable but these were unavailable and two man tents were used in Macedonia. Reserve net supplies for damaged nets were not included in logistics requests and frequently there were operational shortages. Mosquito-proof canvas huts could have been adapted on a larger scale.

In view of these restrictions, and despite concerns over quinine efficacy, military orders prioritized quinine prophylaxis with the aim to interrupt transmission and eliminate the parasite reservoir. It was recognized that quinine treatment could lessen the severity of an infection and shorten the period of early infectivity even though relapses invariably occurred. In the UK, during 1916, the total issue of quinine exceeded 25 tons, or nearly 66 million 5-gr doses [14], and because of supply issues the Government requisitioned all quinine stocks. The average monthly supply in 1917 and 1918 was 5.5 tons. The influence of Ross on this strategy was substantial [78, 89]. Some doctors, however, thought that in regions of Macedonia, Albania, and the Tauris Mountains there were different forms of malaria that could not be treated by quinine [76]. This undermined confidence in its use although there could be no dilemma in drug choice as no alternative was available. In German and Austrian publications unrestricted prophylaxis was promoted and its use was a military order [76].

Because of restrictions on supplies there was increased interest in Germany in manufacturing a synthetic alternative anti-malarial, although the chemical industry was more committed to developing poison gases [46]. It was only at the end of the War, when Germany was cut-off from the world supply of quinine, that Pamaquin development was stimulated and achieved by 1925 [71]. Questions of resistance to quinine became topical in view of the frequency of recurrences which lead to conflict in Germany on the distinction between acquired drug resistance, and drug selection of pre-existing resistant strains [76, 90].

### Quininization

Quinine prophylaxis was defined by Ross as 'treatment of persons who have not yet shown signs of illness but may possibly become infected’ [78], and by Treadgold as 'regular administration of quinine with a view to prevent the development or recrudescence of clinical malaria’ [80]. The first definition suggests it prevents new infections occurring, whereas the second emphasizes the prevention of recurrent infection due to failed primary clearance. This difference encapsulates the confusion and controversy that surrounded quinine treatment and prophylaxis at the time of the First World War. This was fuelled by widely varying practices and published recommendations in dosage and regimens which were mostly empirical, and subsequently were directed by military authorities following quinine trials in the UK, France, Macedonia, and Palestine (Table 6). Often trials did not assess the percentage not infected before prophylaxis commenced. Results on efficacy were variously reported as good or poor by different national authorities. Quinine was widely used by the German troops but data was censored on its evaluation, [68, 85, 91].

**Table 6:** **British and German military quinine prophylactic and treatment regimens for malaria 1914–1918**

Author or source [reference]	Area and subjects	Year	Prophylactic dose regimen ^a,b^	Treatment dose regimen
British				
Hehir [79]	India	1914	All troops quinine treated not < 4 months	–
Wenyon [17]	Macedonia	1916	5,10,20,30gr/day tried	30gr/day x 24 days + arsenic + iron
		1918		
Editorial BMJ [81]	No restriction	1916	Large doses needed	–
Laveran [92]	Macedonia	1917	40gr/day (split dose) x 7.5 months	–
Treadgold [80]	Macedonia	1917	5-15gr x 2 consecutive days at 1 wk intervals	–
Manson [93]	No restriction	1917	5gr/day, or 10gr/x2wk, or 15gr x 10th day	5gr x 3 days, or 15gr x1 dose x 1 wk; Iron + arsenic for 8wks; then 5gr/day x 3 months x every Spring x 2 years
Stephens [63]	Liverpool evacuated troops	1917	Interrupted courses preferable to continuous. Various doses and scheduling. Arsenic not beneficial, strychnine more beneficial	–
Ross [94]	UK special treatment centres	1917	5-60gr/day investigated x 3 wks in various schedules and administration routes. Concluded 60gr/wk reduces relapses to 10% per month	Short and long sterilizing treatments. No difference with administration route
Regulations UK Govt [95]	UK troops	1917	**Y scheme**: Every soldier to keep 60gr for self-treatment if relapses. Report relapse day or night for blood film and fever check	**Y scheme**: 15gr/day x 14 days then 60gr/wk x 6 days in optional divided doses
Paisseau [96]	Balkans	1917	1-2gr/day x 3 days x 1–2 months Iron + arsenic x 1–2 months	IV adrenaline if rigors 2gr preferred if *P.falciparum*
		1918		
Anderson [97]	Macedonia	1917	20-45gr both intensive and interrupted x 1–2 months	IV strychnine and adrenaline
		1918		
Alport [98]	Salonika	1917	1-10gr/day continuous; or 35gr/day x 71 days	Cerebral malaria: 60-100gr in 12 hours x 3 days IM,IV, rectal
War Govt Medical Services Publication [13]	Conflict zones	1914	10gr x 21 days. Arsenic to reduce anaemia with iron and strychnine (IV if severe). Opium (Warburg’s tincture). Venepuncture to reduce toxaemia (≥1 pint), and replace with normal saline^c^	Consider blood transfusion twice fortnightly. Splenectomy in severe anaemia
		1918		
German^d^				
Bruns [91]	Macedonia	1917	0.3 -0.6 g/day + arsenic. Recommended 1.5 – 2.0 g/day x 2 weekly x 6–8 wks. Repeat annually for 3 years	–
		1918		
Kestner [85]	Macedonia	1916	0.3 -0.9 g/day	–
	Romania	1918		
Stendel [99]	Eastern front	1916	0.3 g alternating days x 6 wks	–
Abstracts [100]	No restriction	1918	Summaries on quinine studies. Various doses^e^	–

^a^gr refers to grains of quinine. One grain = 64.8 mg quinine; IM: intra-muscular; IV: Intravenous.

^b^The modern treatment regimen is 20 mg/kg loading dose, and then 10 mg/kg x 3 daily x 7 days. This total loading dose in grains for a 75 kg man, equates to 23.1 grains. The maintenance dose equates to 11.6 grains x 3 daily.

^c^Needs further investigation.

^d^German studies use dose suffix of g (not gr).

^e^Details may have been censored.

Although quinine prophylaxis was often ordered it was not enforced and officers were neglectful [18]. Ross considered that therapeutic efficacy in a war setting related to the level of supervision of intake preferring long-term daily prescription which was difficult to implement. He differed from several other European malariologists in recommending continuous dosage [78]. Quinine parades with supervised observation interrupted activities and falls in attendance were difficult to correct if 100,000 troops were involved [1]. A great amount of preparation was required to make this strategy effective. During the First World War, *P. vivax* malaria showed a pattern of relapse within two months of initial treatment with a tendency to relapse about 8–10 months later, but it was unknown whether recurrences were recrudescent blood stage infection or derived from a latent tissue stage [101]. Ross later changed his opinion and conceded that some relapses occurred in spite of the most vigorous daily supervision, but disagreed with the French whose approach was to suspend quinine between relapses [89]. Memoranda from the British Ministry of War endeavoured to provide some clarity [10]. Initially orders were given from 1st June 1916 for daily doses of 6 gr quinine to troops in malarial areas following an attack of malaria, but as summer progressed, all troops, whether malarial or not, were given quinine as there was some evidence it mitigated the severity of an attack. The fundamental problem related to the knowledge gap concerning the exo-erythrocytic hepatic stage of development of *P. vivax.* Without this knowledge it was impossible for clinicians to distinguish between the need for a drug to provide a radical cure of *P. vivax* infection which cleared hepatic hypnozoites, from suppressive cure, which would control overt clinical attacks of both *P. vivax* and *P. falciparum* malaria [102]. As a result the war-time data on a large number of clinical trials from centres in the UK aiming to evaluate quinine, and routes of its administration, were difficult to interpret [63, 94]. This dilemma was captured at a meeting of the Royal Society of Tropical Medicine and Hygiene held in London in late 1917 when, following the presentation of trial results by Ross comparing treatment regimens, two directly opposing opinions were expressed [103]. Sir William Osler emphasized that 'the man who could not treat malaria successfully with quinine should give up the practice of medicine’ , then adding 'in divided doses’. In contrast, Warrington-Yorke commented that 'no-one is able to state how quinine should be taken, in which manner, and in what doses’. He considered the results presented by Ross meaningless as all treated cases were not diagnosed following microscopic confirmation of malaria at the time of relapse. In conclusion, without definite evidence on efficacy the data was 'worse than useless, because it misleads other people and so prevents progress’ [103]. Data on the therapeutic value of iron, arsenic and strychnine which were sometimes used as adjunct treatments (Table 6) were not systematically assessed. A few small clinical trials that investigated treatment with and without arsenic came to varied conclusions [63, 104]. Revised recommendations called the Y scheme outlined in Table 6 were eventually implemented by the British.

There were concerns related to possible effects of quinine in reducing immunity to severe malaria, or advantages if released malarial antigen stimulated immunity [105]. Theories on malaria toxins lead some practice to use venipuncture to remove the toxic substance, which had echoes of enlightenment blood-letting [13] (Table 6). It is unclear from the government publication mentioning this how frequently it was practised, or at what stage during the War. No really satisfactory result was obtained. No one treatment provided a certain cure and the most effective treatments, and those with multiple intra-muscular quinine injections, were almost more severe than the disease. The use of the maximum tolerated dose instead of the minimum effective dose was preferred in favour of reducing the risk of selecting resistant parasites. Some considered quinine was a useful prophylactic against Blackwater Fever, and it was also used for the treatment of pneumonia [106]. Conversely Treadgold concluded that the severity of malaria in Macedonia was increased in some cases by prior quininization. He quoted Celli who claimed quinine prophylaxis produced some effect, but compared to results of mosquito protection, the results were negligible [80].

### Lest we forget

The First World War was a vast uncontrolled experiment in politics, military innovation and medical practice. Malaria parasites were unaligned and unexpected adversaries which battered both the victors and the vanquished. Their control would require breaching political barriers as well as improved scientific knowledge and understanding. Medical progress would require much greater insight into experimental method and the complex nature of the adversary, the individual, and the environmental circumstances of war [107]. At the beginning of the War the extent of the malaria problem was effectively unknown to military planners and as a consequence there were insignificant efforts at containment. Malaria rapidly spread to civilian populations as well as to the military, creating a complex emergency. In temperate zones, where *P. vivax* was the dominant parasite, ideal conditions were developing for parasite and mosquito, possibly enhanced by the climatic conditions of the strong 1914–1915 and 1918–1919 El Nino events. The trans-migration of troops and parasites across Europe favoured the spread of new parasite strains, which flourished in non-immune host populations of soldiers. Textbook ideal conditions were created for multiple epidemics, which would involve vast numbers of soldiers, from South–East England to the shores of Arabia, from the Arctic to the Mediterranean. Moreover, the statistical estimates of the numbers of malaria cases derived in this analysis are likely to be sizable underestimates, not least because malaria surveillance data went un-reported from several war fronts, as did civilian related malarial deaths. Censorship of German data limited information available for axis forces on case numbers, mortality, as well as on their military and medical response.

The military and medical response of allied forces was at first limited, perhaps because of perceived greater demands for directed military action the response gathered pace in 1916 when it was understood that whole armies could be immobilized by the malaria parasite, defeating military objectives. Medical facilities were required on a very large scale to treat acutely affected and chronically disabled soldiers with recurrent symptoms. In the search for scientific evidence this became an experimental field of medicine with research on drug trials, haematology and immunology, including attempts to culture the malaria parasite [108–110]. The priority for an effective treatment was paramount but, in the absence of knowledge of the exo-erythrocytic developmental stage of *P. vivax,* these attempts would lack resolution. Not until the late 1920s was it apparent that *P. vivax* recurrences followed a different temporal pattern to *P. falciparum* [101]. Prior to then the treatment response to the two parasitic species was frequently viewed as synonomous. The endeavour to defeat this enemy became controversial despite the efforts of Ross to co-ordinate treatment trials and it was not resolved until years after the War. It is a good example of the failure of the clinical trial approach to resolve treatment practices when the scientific mechanisms underlying the disease are unknown. The need for patient care of soldiers was paramount and in response, both in Liverpool and Sierra Leone, the Liverpool School of Tropical Medicine closed its research laboratories and converted them to hospital facilities that could accommodate clinical trials [62]. Grassi in Italy came out of retirement in 1917 to aid the Italian effort to cope with the malaria crisis by running a dispensary and research station [6]. The influence of Ross on prevention guided sanitation efforts and implementation of rapid malaria surveys, which included entomological investigations, which acted as a platform for control activities and aided the allied military effort. The lower reported number of allied compared to Turkish malaria deaths in Macedonia probably reflects better allied malaria control strategy. These efforts served to educate soldiers and officers on the malaria problem, which was no longer hidden.

**Figure 12:**
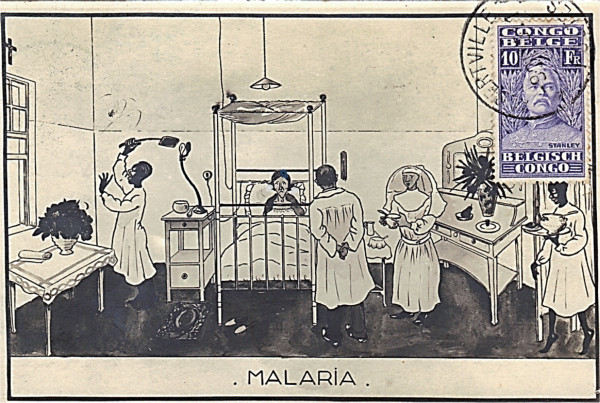
**Post-war postcard illustrating combination management of a malaria patient.**

Underlying national and international conflicts influenced progress. These varied from opinions on treatment such as 'there are almost as many treatments of malaria as there are people who have written on the subject’ [103], to disagreements on ideas for bed net design [17], or more fundamentally, to differences in racial theories of immunity to malaria [76]. The culture of command was an umbrella over-riding alternative choices, and recommendations were difficult to reach by Committees whose agendas were broad, and at times imperialistic [64, 111]. Much credit rests on individual medical officers whose clinical acumen and decision-making was paramount in patient care in rapidly changing local circumstances. Patients could be bewildered by the variety of treatment options as illustrated in this post-war cartoon postcard from sub-Saharan Africa (Figure 12). Allied activities become better integrated later in the War as demonstrated by the rapid control of the English outbreak. The nationalization of malaria control commenced rapidly after the War in those European countries most affected, Greece [5], Italy [6], and Palestine [112], but with little progress in Africa. The disease remains a threat in war zones today [113], and on the Centenary of the First World War, soldiers continue to experience high infection rates in malarious areas in Africa [114–116].

James in his Presidential address to the Royal Society of Tropical Medicine in London concluded that 'everyone who had actually taken part in efforts to deal with malaria in different parts of the world during the War came home with the uncomfortable feeling that we knew much less about the disease than we thought we did, and that it might be quite a good plan to sink our pride and to begin again, in all humility, and with greater respect and reverence, to try to fathom out some of its mysteries’ [117]. A view that is as relevant today.
